# Simulating federated learning for steatosis detection using ultrasound images

**DOI:** 10.1038/s41598-024-63969-x

**Published:** 2024-06-10

**Authors:** Yue Qi, Pedro Vianna, Alexandre Cadrin-Chênevert, Katleen Blanchet, Emmanuel Montagnon, Eugene Belilovsky, Guy Wolf, Louis-Antoine Mullie, Guy Cloutier, Michaël Chassé, An Tang

**Affiliations:** 1 Centre de Recherche du Centre Hospitalier de l’Université de Montréal (CRCHUM), Montréal, QC Canada; 2 Institute of Biomedical Engineering, Université de Montréal, Montréal, QC Canada; 3 Laboratory of Biorheology and Medical Ultrasonics - CRCHUM, Montréal, QC Canada; 4 Radiology, Radiation Oncology and Nuclear Medicine Department, Université de Montréal, Montréal, QC Canada; 5 CISSS de Lanaudière, Joliette, QC Canada; 6 Clinical Laboratory of Image Processing - CRCHUM, Montréal, QC Canada; 7 Mila – Quebec Artificial Intelligence Institute, Montréal, QC Canada; 8 Concordia University, Montréal, QC Canada; 9 Department of Mathematics and Statistics, Université de Montréal, Montréal, QC Canada; 10 Department of Medicine, Division of Critical Care Medicine, Centre Hospitalier de l’Université de Montréal (CHUM), Montréal, QC Canada; 11 Faculty of Medicine, Université de Montréal, Montréal, QC Canada; 12 Département de Radiologie, Centre Hospitalier de l’Université de Montréal (CHUM), 1058 Rue Saint-Denis, Montréal, QC H2X 3J4 Canada

**Keywords:** Steatosis, B-mode ultrasound image, Federated learning, Data partition, Class imbalance, Ultrasound, Machine learning, Non-alcoholic fatty liver disease

## Abstract

We aimed to implement four data partitioning strategies evaluated with four federated learning (FL) algorithms and investigate the impact of data distribution on FL model performance in detecting steatosis using B-mode US images*.* A private dataset (153 patients; 1530 images) and a public dataset (55 patient; 550 images) were included in this retrospective study. The datasets contained patients with metabolic dysfunction-associated fatty liver disease (MAFLD) with biopsy-proven steatosis grades and control individuals without steatosis. We employed four data partitioning strategies to simulate FL scenarios and we assessed four FL algorithms. We investigated the impact of class imbalance and the mismatch between the global and local data distributions on the learning outcome. Classification performance was assessed with area under the receiver operating characteristic curve (AUC) on a separate test set. AUCs were 0.93 (95% CI 0.92, 0.94) for source-based partitioning scenario with FedAvg, 0.90 (95% CI 0.89, 0.91) for a centralized model, and 0.83 (95% CI 0.81, 0.85) for a model trained in a single-center scenario. When data was perfectly balanced on the global level and each site had an identical data distribution, the model yielded an AUC of 0.90 (95% CI 0.88, 0.92). When each site contained data exclusively from one single class, irrespective of the global data distribution, the AUC fell in the range of 0.34–0.70. FL applied to B-mode US images provide performance comparable to a centralized model and higher than single-center scenario. Global data imbalance and local data heterogeneity influenced the learning outcome.

## Introduction

Metabolic dysfunction-associated fatty liver disease (MAFLD) is a prevalent liver condition^1–3^ characterized by the presence of steatosis, the accumulation of fat in over 5% of hepatocytes^4^. Individuals with diabetes mellitus are particularly at risk, as MAFLD is associated with an increased overall mortality rate in these patients^5^. Therefore, the accurate detection of hepatic steatosis plays a pivotal role in delivering appropriate patient care. While liver biopsy serves as the reference standard for grading hepatic steatosis^6^, its high cost and invasive nature prevent its use for large-scale screening or surveillance. The development of a noninvasive diagnostic method to detect steatosis is crucial for reducing the reliance on biopsies and its associated complications.

Recently, there has been a significant focus on leveraging machine learning to detect and classify liver diseases^7–10^. Classic machine learning models including support vector machines, k-nearest neighbors and tree models were employed for detecting and classifying steatosis using ultrasound images^11,12^. However, the performance of these models is heavily reliant on feature selection techniques. Deep learning architectures have demonstrated the ability to learn ultrasound imaging features and accurately classify the severity of chronic liver disease. Steatosis assessment using ultrasound images has demonstrated the superiority of deep learning-based approaches over hepatorenal index method and gray-level co-occurrence matrix algorithm^4^. Notably, convolutional neural network models using B-mode US examinations^13^ have shown great potential for detection and grading of liver steatosis in a single-center setting^10^.

Machine learning models trained on diversified datasets combining data from multiple institutions can improve generalizability. To address concerns related to data privacy and regulation, federated learning (FL)^14,15^ has emerged as a potential method for training machine learning models across multiple clinical sites. FL has garnered interest, particularly in the medical domain, as it involves the exchange of trained models instead of raw training data among multiple institutions. This approach enhances the protection of patient privacy while allowing for collaborative model training. With large datasets, it may be cost prohibitive to transfer data between sites and sharing weights between sites may be the optimal approach. However, it is currently unclear how data distribution, data quantity, partitioning across sites, clients, centers, or hospitals (which we will refer to from now on as sites), and choice of FL algorithms impact the model performance. A systematic assessment of the impact of various data distributions would help inform the use of various FL algorithms for clinical use.

The purpose of this study was to implement four data partitioning strategies and to evaluate the performance of four different FL algorithms for each data partitioning strategy for the detection of hepatic steatosis using deep learning on B-mode US images. The impact of data imbalance and the mismatch between the global and local data distribution on the model's performance were also examined.

## Materials and methods

### Study design

This retrospective, cross-sectional, case-control, diagnostic, dual-center, model creation study was approved by the institutional review board of the Centre Hospitalier de l’Université de Montréal (CHUM), Québec, Canada. All the methods performed in this study comply with all relevant ethical guidelines and regulations. Requirement for informed consent from all patients and/or their legal guardian(s) was waived for the private dataset from our institution by the institutional review board of the Centre Hospitalier de l’Université de Montréal (CHUM) due to the retrospective nature of the study. The public dataset is available online through the Creative Commons Attribution licensing^16^. In this study, we focused on a binary classification task, *i.e.,* to classify steatosis grade S0 vs S1 or higher, which correspond to class or label (which we will refer to from now on as class) S0 and ≥ S1 respectively.

### Datasets

Two datasets collected at separate sites were used in the current study: a publicly available dataset acquired in the Medical University of Warsaw, Poland^4,16^ and a private dataset from the Centre Hospitalier de l’Université de Montréal (CHUM). The ultrasound images in the public dataset were acquired using the GE Vivid E9 Ultrasound System (GE Healthcare INC, Horten, Norway) equipped with a sector probe operating at 2.5 MHz. The settings followed the default general abdominal preset with harmonic imaging. Patients in the public dataset were included based on these criteria: severely obese, undergoing abdominal ultrasound, with a wedge liver biopsy performed during bariatric surgery available for histopathological assessment^4,16^. The B-mode abdominal US images within the private dataset originated from seven different scanners, including iU22 (Philips), Aplio 500 and i800 (Canon Medical Systems), Acuson S2000 and S3000 (Siemens Healthineers), Sequoia (Siemens Healthineers), and LOGIQ E9 (GE HealthCare). These images were acquired following the institutional clinical US protocol. Patients were included in the private dataset based on these criteria: availability of B-mode abdominal US and liver biopsy within 1 year of each other and a histopathological diagnosis of MAFLD, metabolic dysfunction-associated steatohepatitis (MASH), or MASH-related cirrhosis and excluded if they had any other causes of chronic liver disease. Patients were excluded if more than one biopsy result was available, they had fewer than 10 images available, or images were deemed of poor quality.

The private dataset included in the current study was published in a prior study^10^. The prior study reported the performance of a deep learning model trained on dataset from a single institution and compared the performance of the deep learning model with that of 6 human readers for detection and grading of liver steatosis.

**Figure 1:**
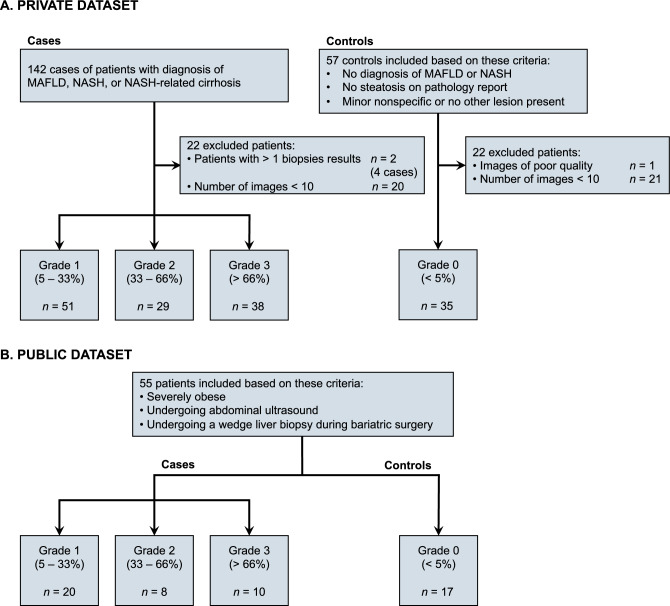
Flowchart of patient selection: (**a**) private dataset and (**b**) public dataset^4^. *MAFLD* metabolic dysfunction-associated fatty liver disease, *NASH* nonalcoholic steatohepatitis.

The characteristics of the 208 patients from two sites included in this study are summarized in Table 1. The public and private datasets respectively included 55 (mean age ± standard deviation: 40 ± 9, 20% men) and 153 patients (52 ± 13, 49% men). The percentage of female patients in the public dataset is higher (80%) compared to the private dataset (51%). The mean BMI of the patients in the public dataset exceeds that of the private dataset by 15.1 kg/m^2^. The steatosis grades distributions in the two datasets are similar. Flowchart of patient selection is shown in Fig. 1. Patient identifiers were encrypted using salt and pepper cryptographic hashing in the private dataset. Nominal information was removed from the images.

**Table 1:** Characteristics of the 208 patients from two centers included in this study.

Characteristic	Private dataset	Public dataset
Sex		
Male	75/153 (49%)	11/55 (20%)
Female	78/153 (51%)	44/55 (80%)
Age (years)		
Mean ± SD	52 ± 13	40 ± 9
Median (range)	54 (20–79)	
IQR	43–62	
BMI (kg/m^2^)		
Mean ± SD	30.8 ± 8.0	45.9 ± 5.6
Time between exam and biopsy (days)		
Mean ± SD	75.3 ± 108.4	
Median (range)	14 (0–364)	(1–2)
IQR	1–140	
Steatosis grade		
0 (< 5% hepatocytes involved)	35/153 (23%)	17/55 (31%)
1 (5% – 33% hepatocytes involved)	51/153 (33%)	20/55 (36%)
2 (33% – 66% hepatocytes involved)	29/153 (19%)	8/55 (15%)
3 (> 66% hepatocytes involved)	38/153 (25%)	10/55 (18%)

*BMI* body-mass index, *IQR* interquartile range, *SD* standard deviation.

### Image selection

**Figure 2:**
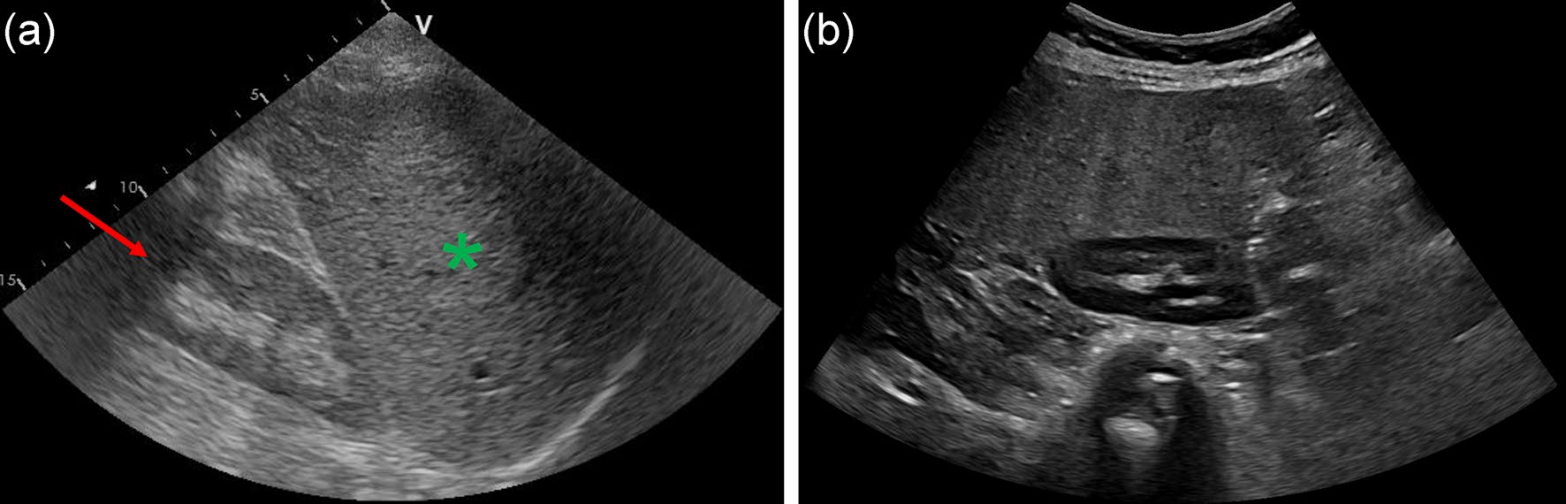
Example of representative B-mode ultrasound images from the public and private datasets. (**a**) Steatosis grade 0 (3% hepatocytes with steatosis) from the public dataset shows the liver (asterisk) and right kidney (arrow). (**b**) 73-year-old female with non-alcoholic steatohepatitis with steatosis grade 3 from the private dataset shows moderate activity at the stage of cirrhosis.

Ten B-mode ultrasound images were randomly selected for each patient. The original images were in digital imaging and communications in medicine (DICOM) format. Prior to being transferred to our study, all images were de-identified. The images had a resolution of 434 × 636^4^ in the public dataset and typically 960 × 1280 pixels in the private dataset. The images in the datasets were cropped at the center to eliminate irrelevant information that could potentially impede the training process. Representative examples from both the public and private datasets are shown in Fig. 2.

### Test set creation

The test set was created as follows: 10 patients were chosen from both the public and private datasets. Out of these 10 patients, 5 were randomly chosen from those with steatosis, while the remaining 5 were randomly selected from patients without steatosis. This distribution ensures that the classes in the test set are uniformly represented. The remaining data from both the public and private datasets were combined to form the training set. Various data partitioning strategies (as detailed later) were applied to this training set to construct a realistic federated learning dataset.

### Reference standard

**Figure 3:**
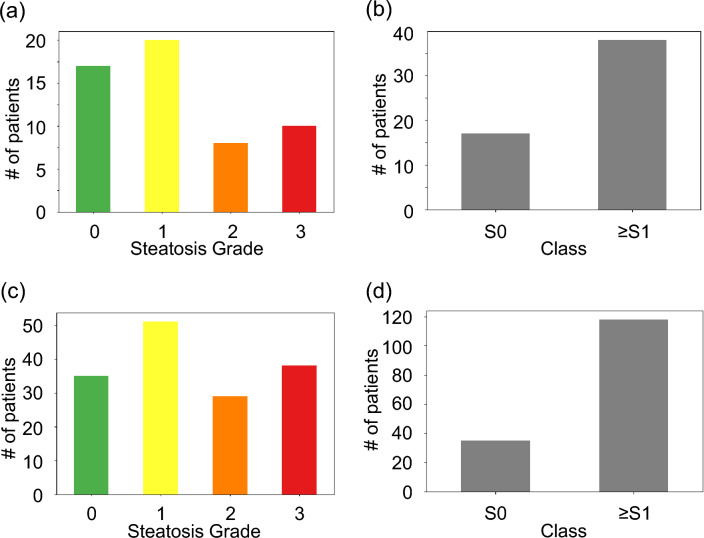
Steatosis grades and class distributions among patients in the public dataset (**a**–**b**) and the private dataset (**c**–**d**).

The reference standard in the two datasets was histopathological assessment of liver biopsies graded with the same four-point ordinal score according to the percentage of hepatocytes containing macrovesicular fat: grade 0 (normal or < 5%), grade 1 (mild or 5–33%), grade 2 (moderate or 33–66%), and grade 3 (severe or > 66%). The histopathological assessment was performed according to the NASH Clinical Research Network scoring system by a single pathologist for the public dataset and from clinical interpretations by pathologists for the private dataset^4,10, 16^. Figure 3 illustrates the distribution of patients according to their steatosis grade and training classes, encompassing both the public and private datasets.

### Simulation environment and configuration

To create a simulated FL environment, we employed three virtual computers, each equipped with an NVIDIA GRID V100-16Q GPU. These virtual systems formed the core infrastructure for executing the Flower FL framework^17^, which allowed us to establish one server and two sites. The structure of our FL simulation environment is illustrated in Supplementary Figure S1.

We used a VGG16-based model^18^, known for its effectiveness in detecting steatosis on B-mode ultrasound images^10^. The input and output layers have the shape of (128, 128, 3) and (1), respectively. The model’s initial weights were randomly drawn from a uniform distribution. Note that each site’s model shared identical initial weights. Ultrasound images were resized to 128 × 128 pixels while preserving the original aspect ratio by zero padding. Image intensities were normalized to a range of 0 to 1. To enhance the model's resilience, simple data augmentation techniques, such as horizontal flip and rotation, were employed. The batch size was set to 32 and Adam was chosen as the optimizer. To address class imbalance issue, the focal loss, commonly used in object detection models, was selected as the loss function. We trained the model using either 1 local epoch for 100 rounds or 5 local epochs for 20 rounds (unless specified otherwise). We conducted each experiment three times for accurate analysis.

Software was developed in Python (version 3.8; Python Software Foundation, Wilmington, United States) with albumentations (version 1.3.1), flwr (version 1.5.0), matplotlib (version 3.7.2), numpy (version 1.23.5), opencv_python_headless (version 4.8.0.76), pandas (version 2.0.3), Pillow (version 10.0.1), PyYAML (version 6.0.1), scikit_learn (version 1.3.0), seaborn (version 0.13.0), and tensorflow (version 2.12.0).

### Data partition strategies

A key challenge in federated learning is the heterogeneity of data distributions across participating sites. We aim to construct a representative and comprehensive non-independent and identically distributed (IID) data setting that adequately encompasses various non-IID scenarios in federated learning. Multiple data partition strategies were employed to create non-IID data within each site, thereby simulating a realistic FL dataset.

#### Class distribution skew

We investigated two types of class distribution skew, namely distribution-based and quantity-based class distribution skew^19^.

- *Distribution-based class distribution skew*: this partition strategy uses the Dirichlet distribution^20^ to allocate a proportion of the data for each class within the population. The Dirichlet distribution is widely used in simulating FL data and can be denoted as *Dir*(*β*), where *β* represents the concentration parameter, regulating the degree of class imbalance.
- *Quantity-based class distribution skew*: each site contains data of a fixed number of classes. The data belonging to each class are equally partitioned into the sites that own this class. We deliberately constructed an extreme scenario in which each site exclusively possessed data from a single class to highlight the effect of quantity-based class distribution skew.

#### Data quantity skew

Data quantity skew refers to the uneven distribution of data across different sites^19^. The Dirichlet distribution *Dir*(*β*) can also be employed to allocate varying amounts of data to each site. In this case, the concentration parameter *β* determines the degree of imbalance in data quantities across sites.

#### Source-based partition

Given that the data used in this study originates from two distinct sites, it is natural to divide the data based on its source into the respective simulated sites. For example, we can allocate all the public data to site 1, while assigning all the private data to site 2. This particular scenario closely mirrors the real-world FL setup.

### Federated learning algorithms

Four different FL algorithms were evaluated to assess their effectiveness in training a resilient model under non-IID data setting. The training process of FL algorithms is described below.

*FedAvg*^15^: the training process of FedAvg is depicted in Supplementary Figure S2. In each training round *t*, the server first distributes the weights of the global model from the previous round *t*−1, ${\omega }^{t-1}$, to each participating site. Each site then independently trains the model with its local data for *k* epoch(s). Next, each site transmits the weights of their trained model, ${\omega }_{i}^{t-1}$ back to the server. Finally, the server aggregates the received weights using Eq. (1) to obtain the updated global model weights, ${\omega }^{t}$.

1 $$\omega^{t} = \mathop \sum \limits_{i = 1}^{N} \frac{{n_{i} }}{n}\omega_{i}^{t - 1}$$

where *N* represents the total number of sites, *n* denotes the overall number of samples in the population and ${n}_{i}$ indicates the number of samples at site *i*.

*FedAvgM*^21^: this algorithm incorporates server momentum into the original FedAvg framework by leveraging a running accumulation of the gradient history. This modification has shown significant performance improvements over FedAvg on non-IID data^21^. The update process of the global model weights, ${\omega }^{t}$ can be summarized by Eqs. (2)–(4).

2 $$\Delta_{t} = \omega^{t - 1} - \omega_{FedAvg}^{t}$$

3 $$\upsilon^{t} = \beta \upsilon^{t - 1} + \Delta_{t}$$

4 $$\omega^{t} = \omega^{t - 1} - \eta \upsilon^{t}$$

where ${\omega }^{t-1}$ is the global model weights after training round *t*−1, ${\omega }_{FedAvg}^{t}$ denotes the aggregated weights by FedAvg, $\beta$ represents the server momentum and $\eta$ is server learning rate.

*FedYogi*^22^: this variation of the FedAvg algorithm incorporates an adaptive optimization technique. This algorithm offers the advantage of easy tuning, as it allows for a wide range of hyperparameter values that can yield optimal performance. The procedure for updating the global model weights, ${\omega }^{t}$ can be summarized using Eqs. (5)–(8).

5 $$\Delta_{t} = \omega_{FedAvg}^{t} - \omega^{t - 1}$$

6 $$m^{t} = \beta_{1} m^{t - 1} + \left( {1 - \beta_{1} } \right)\Delta_{t}$$

7 $$\upsilon^{t} = \upsilon^{t - 1} - \left( {1 - \beta_{2} } \right)\Delta_{t}^{2} sign\left( {\upsilon^{t - 1} - \Delta_{t}^{2} } \right)$$

8 $$\omega^{t} = \omega^{t - 1} + \eta \frac{{m^{t} }}{{\sqrt {\upsilon^{t} } + \tau }}$$

where ${\omega }_{FedAvg}^{t}$ denotes the aggregated weights by FedAvg, ${\omega }^{t-1}$ is the global model weights after training round *t−*1, ${\beta }_{1}$ denotes the momentum parameter, ${\beta }_{2}$ is the second momentum parameter, $\eta$ represents the server side learning rate, and $\tau$ controls the degree of adaptability.

*FedProx*^23^: this algorithm alters the local objective function by introducing an ${L}_{2}$ regularization term, as shown in Eq. (9).

9 $$L^{\prime } = L + \frac{\mu }{2}\left\| {\omega - \omega^{t - 1} } \right\|^{2}$$

where $L$ is the original objective function, $\mu$ controls the weight of regularization, $\omega$ and ${\omega }^{t-1}$ are the local model weights and current global model weights. The ${L}_{2}$ regularization term restricts the distance between the local and global model. This modification encourages the aggregated model to be closer to the global optimum.

The FL models were trained with 1 and 5 local epochs, aligning with prior study^21^. For comparison, we trained a centralized model by combining the public and private datasets into a single dataset. Furthermore, we simulated a scenario resembling a single-center study by training a model exclusively using the private data.

### Data imbalance

Global and local class imbalances^24,25^ are two prevalent issues encountered in real-world FL problems. A significant disparity often exists between the imbalances observed locally and globally. For instance, a class that is predominant in a local subset may be a minority in the overall population, which can considerably influence the learning outcomes.

#### Global class imbalance

To simulate the phenomenon of global class imbalance, we generated datasets of the same size but with varying percentages of data belonging to a designated class. For instance, we can manipulate the percentage of data belonging to class S0 to represent 10% of the dataset.

#### Mismatch between global and local data distribution

To introduce heterogeneity in the local data distributions^25^, we used the following approach: Let $\alpha \in \left[0, 1\right]$ determine the level of local data homogeneity. We assign each site a portion of $\alpha$ IID data. The local class distribution of site *i* is a combination of the global class distribution and the Dirac distribution of a fixed specific class ${c}_{i}$. The class distribution of site *i*, denoted as ${P}_{i}$, can thus be written as:

10 $$P_{i} = \alpha P_{global} + \left( {1 - \alpha } \right)\delta_{{c_{i} }}$$

where ${P}_{global}$ represents the global class distribution and ${\delta }_{{c}_{i}}$ denotes the Dirac distribution of class ${c}_{i}$. A value of *α* = 0 indicates that each site possesses data from only one single class, while a value of 1 means that the class distribution for the data at each site is the same as that of the global data.

We employ the weighted average cosine similarity, denoted as $\overline{CS}$, to assess the disparity in class distribution between the global and local data at each site, as shown in Eqs. (11)–(12).

11 $$CS_{i} = \frac{{\upsilon_{i} \cdot V}}{{\left\| {\upsilon_{i} } \right\|\left\| V \right\|}}$$

12 $$\overline{CS} = \mathop \sum \limits_{i = 1}^{N} \frac{{n_{i} }}{n}CS_{i}$$

where ${CS}_{i}$ is cosine similarity of the class distribution between the overall population and site *i*, ${\upsilon }_{i}=\left[{n}_{i}^{0}, {n}_{i}^{1}...\right]$ is a vector that denotes the number of samples of each class at site *i* and $V=\left[{\sum }_{i=1}^{N}{n}_{i}^{0}, {\sum }_{i=1}^{N}{n}_{i}^{1}...\right]$ denotes the composition of the population. *N* represents the total number of sites, *n* denotes the overall number of samples in the population, ${n}_{i}$ indicates the number of samples at site *i*. A $\overline{CS}$ value of 1 indicates a perfect match in the class distribution between the global data and local data at each site. A lower value signifies a mismatch in the class distribution between the global and local data.

To investigate the influence of data imbalance, we incorporated two factors: class imbalance at a global level and data heterogeneity at a local level. At the global level, we generated datasets with a fixed size of 84 patients, but with varying proportions of data belonging to class S0 (ranging from 10 to 50%). We then partitioned the global data into different sites using Eq. (10). To create varying levels of local data heterogeneity, we assigned a range of values to the parameter, *α* that determines the level of local data homogeneity (*i.e.,* 0, 0.2, 0.4, 0.6, 0.8, and 1).

### Statistical analysis

Descriptive statistics included the exact count and percentages. Cosine similarity was used to quantify the disparity in class distribution between global and local data. The performance of models was evaluated by accuracy, sensitivity, specificity, positive predictive value (PPV), negative predictive value (NPV) and area under the receiver operating characteristic curve (AUC). AUC was the preferred metric due to its robustness in mitigating the impact of class imbalance issues. We reported the mean and 95% CIs computed by jackknife. Comparison of AUCs was performed with the DeLong test.

## Results

### Simulated federated learning scenarios

**Figure 4:**
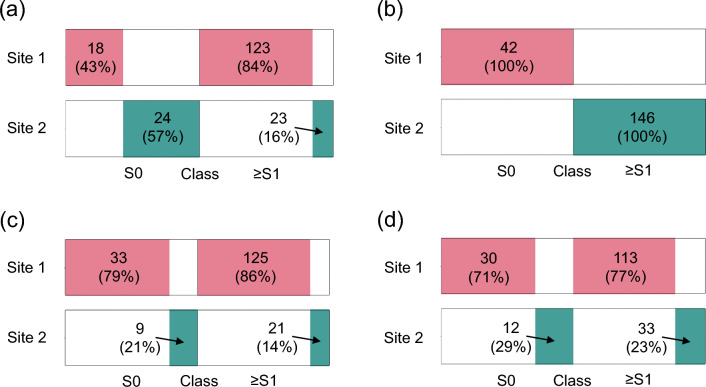
Resulting data distribution from different data partition strategies: (**a**) distribution-based class distribution skew with $\beta =2$, (**b**) quantity-based class distribution skew, where each site exclusively owns data of one class, (**c**) quantity skew with $\beta =2$, and (**d**) source-based partition. The size of the color-filled rectangles represents the percentage of data belonging to a class owned by a site. The number of patients and the percentage belonging to each class at each site are provided.

Figure 4 illustrates the data distribution resulting from different data partition strategies applied to the training data, which combined both the public and private data. The percentage and exact count for each class are indicated in the figure. An extreme scenario was created by quantity-based class distribution skew, where site 1 and site 2 processed all the 42 samples from class S0 and all the 146 samples from class ≥ S1 respectively.

Table 2 presents the performance for the final model, evaluated using the test set under different scenarios and employing various FL algorithms. The models exhibited the lowest performance (AUC ≤ 0.69) in the scenario where each site exclusively held samples from a single class. In other scenarios, FedAvg and FedProx demonstrated higher performance with an AUC ≥ 0.87. Under quantity skew scenario, FedAvg and FedProx achieved AUCs of 0.92 [95% CI 0.91, 0.93] and 0.92 [95% CI 0.90, 0.94] respectively. Sensitivity, specificity, accuracy, PPV and NPV are presented in Supplementary Table S1.

**Table 2:** Diagnostic performance of four federated learning algorithms and four data partition strategies.

Data Partition Strategy	Epoch	FedAvg	FedAvgM	FedYogi	FedProx
Class distribution skew (distribution-based)	1	0.87 (0.82, 0.92)	0.81 (0.79, 0.83)	0.80 (0.71, 0.89)	0.87 (0.79, 0.95)
	5	0.87 (0.85, 0.89)	0.76 (0.61, 0.91)	0.83 (0.78, 0.88)	0.88 (0.83, 0.93)
Class distribution skew (quantity-based)	1	0.60 (0.38, 0.82)	0.50 (0.34, 0.66)	0.60 (0.50, 0.70)	0.69 (0.66, 0.72)
	5	0.57 (0.33, 0.81)	0.58 (0.36, 0.80)	0.58 (0.44, 0.72)	0.55 (0.33, 0.77)
Quantity skew	1	0.92 (0.91, 0.93)	0.82 (0.79, 0.85)	0.78 (0.72, 0.84)	0.92 (0.90, 0.94)
	5	0.89 (0.88, 0.90)	0.73 (0.64, 0.82)	0.82 (0.75, 0.89)	0.87 (0.86, 0.88)
Source-based partition	1	0.93 (0.92, 0.94)	0.81 (0.73, 0.89)	0.80 (0.74, 0.86)	0.88 (0.81, 0.95)
	5	0.88 (0.86, 0.90)	0.74 (0.63, 0.85)	0.83 (0.78, 0.88)	0.87 (0.82, 0.92)

Values reported are the areas under the receiver operating characteristic curve (AUCs) for each algorithm and scenario, with 95% CIs in parentheses.

**Figure 5:**
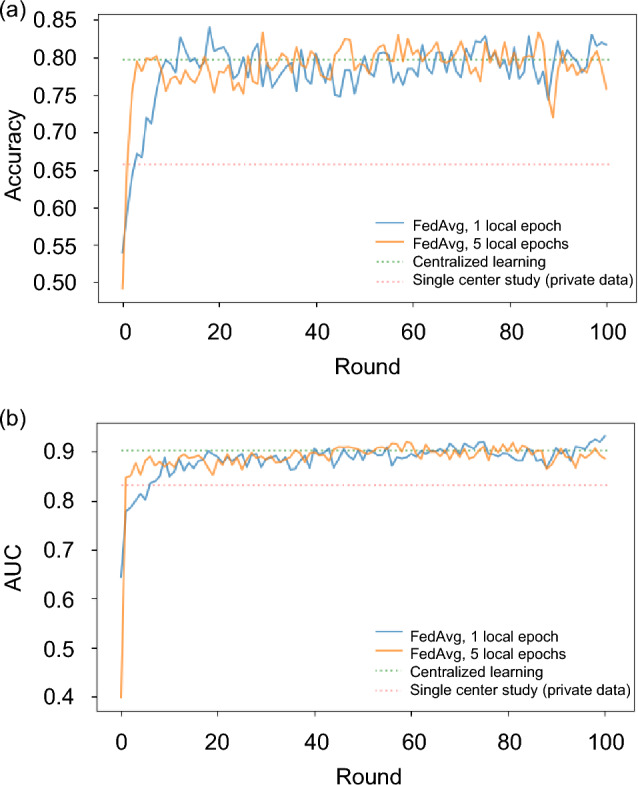
(**a**) Accuracy and (**b**) AUC score, evaluated on the test set for models trained via various approaches: source-based partition scenario, trained with FedAvg and 1 local epoch (blue solid curve); source-based partition scenario, trained with FedAvg and 5 local epochs (orange solid curve); centralized model (green dashed line), model trained solely with private data (red dashed line). *AUC* area under the receiver operating characteristic curve. Note we trained the centralized model by combining the public and private datasets into a single dataset.

Figure 5 illustrates the performance of the global model trained using FedAvg under the source-based partition scenario. The plot showcases the test accuracy and test AUC across different training rounds. Each curve and line represent the average results obtained from three trials. The FL model demonstrated higher performance (AUC of 0.93 [95% CI 0.92, 0.94] for model trained with 1 epoch) compared to the model trained in a single-center study scenario (AUC of 0.83 [95% CI 0.81, 0.85], *P* ≤ 0.001).

### Impact of data imbalance

**Figure 6:**
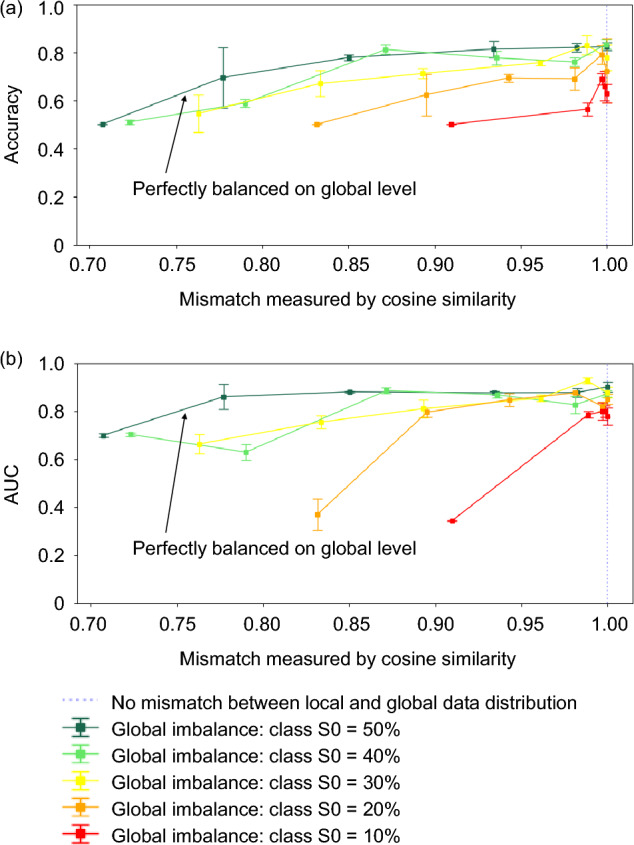
(**a**) Test accuracy and (**b**) AUC scores for models trained under various scenarios characterized by different levels of global class imbalance (ranging from 10 to 50%) and local data heterogeneity. The very left data point on each curve represents the scenario where each site contains data from only one class (extreme case). The model was trained using the FedAvg algorithm with 1 local epoch for 50 rounds. Each experiment was conducted three times. *AUC* area under the receiver operating characteristic curve.

Figure 6 illustrates the performance of the final global model across various levels of global class imbalance and local heterogeneity. The model was evaluated using the test set. We present the test accuracy and test AUC score, plotted against the weighted average cosine similarity as defined in Eq. (12). The model's performance was notably affected by both global data imbalances and local data heterogeneity. The model achieved AUC = 0.90 when the class was perfectly balanced on the global level and each site had an identical data distribution. When each site contained data only from one single class, regardless of the global class distribution, we obtained AUC ≤ 0.70.

## Discussion

Deep learning provides a noninvasive, accurate and automated detection of steatosis. A strength of this approach is its ability to work on existing B-mode US images without the need to purchase dedicated point-of-care US-based devices (e.g., FibroScan) or to purchase additional attenuation software for liver fat quantification. Additionally, federated learning enables training deep learning models on diverse populations while protecting patient privacy. It is thus important to understand factors like data distribution and choice of federated learning algorithms in optimizing model performance.

In this work, we implemented four data partition strategies, evaluated four FL algorithms for each data partitioning strategy, and examined the impact of global class imbalance and local heterogeneity on the learning outcome for the detection of steatosis using B-mode ultrasound images. This study provides insights for improving the performance of FL models in the presence of non-IID data distribution, especially for future studies relying on multi-center data repositories^26^.

Among the four data partition strategies, quantity skew had minimal impact on the learning outcome. This can be attributed to the effective handling of data quantity imbalance by the FedAvg algorithm, which employs weighted averaging during the global model updates. Similarly, Li et al.^19^ found that FedAvg had almost no accuracy loss due to the adoption of the weighted averaging.

The class distribution skew strategy, where each site possessed samples from only one class, posed the most challenging setting. None of the four FL algorithms was successful in training a model with satisfactory performance. This difficulty might arise from the significant disparity between the class distribution of the local and global data. The local optima, which the local models are updated towards during local training, are considerably distant from the global optima. Consequently, the aggregated model may also deviate significantly from the global optima. In^19^, findings indicated when each site only contained data from a single class, the model had the worst accuracy due to the deviation of the local optima from the global optima.

The FL model achieved an AUC that is comparable to the model trained under centralized learning and higher than the model trained in a single-center study scenario. In the context of single-center studies, models trained exclusively on data from a specific site may exhibit strong performance on that site’s data but are susceptible to overfitting. Consequently, their ability to generalize to diverse populations may be limited. In contrast, the model trained under centralized learning setting was trained on a larger and more diverse dataset. This makes it less likely to overfit and allows it to generalize better to unseen examples. However, establishing a centralized learning setting in the real world is challenging due to data privacy concerns. Federated learning addresses those limitations by training on diverse data from multiple sites. By aggregating local models from various sites, federated learning mitigates overfitting and enhances scalability, generalization and privacy. Increasing the number of local epochs during model training in a FL setting could potentially lower the communication cost between the sites and the server. The model trained with more local epochs tended to reach a faster plateau in test accuracy and AUC, requiring fewer training rounds. We observed a reduction in AUC when increasing the local training epochs from 1 to 5. This effect was more pronounced for models trained utilizing FedAvgM. When the local model is trained with a substantial update, involving a large number of local epochs, it can suffer from a drift^27^ in the local updates. This drift may negatively impact the performance of the global model, resulting in unstable convergence, particularly when the data is heterogeneous. Karimireddy et al.^27^ proposed a technique to correct the local update by adding the drift in the local training. However, this doubles the communication size per round.

Our findings on the impact of the data imbalance indicated model performance improved as the data becomes more balanced at the global level. When the number of samples for each class is the same on the global level, the local heterogeneity did not have a significant effect on the learning outcome. However, a notable disparity in model performance, particularly in terms of test accuracy, became evident when the proportion of class S0 is less than 30% at the global level. Specifically, when class S0 = 10%, we encountered difficulty in training a model with satisfactory prediction accuracy, regardless of how the data was partitioned across different sites. Consequently, the test accuracy consistently remained below 0.69 and the AUC always fell below 0.80. On the local level, when each site contained data from only one class, it was difficult to train a satisfactory model, regardless of the level of class imbalance on the global level. In^19^, the performance of FL models for the case where each site only has samples of a single class was examined with the following public image datasets: MNIST, FMNIST, and CIFAR-10. The test accuracy always fell between 9.9% and 40.9%.

Our study has potential limitations. When addressing a different clinical use scenario or using a different type of data, the findings may diverge from those of this study. However, the objective of this paper was to establish a framework and provide guidance for researchers investigating similar problems. While FL aims to provide data privacy by keeping the data locally on the sites, it does not provide a guarantee of privacy because the exchange of model weights between the server and sites may theoretically still expose sensitive information. To provide quantifiable bounds on the amount of allowable disclosure, incorporating differential privacy techniques is required^28,29^. It may be necessary to define a parameter that captures both the global and local data imbalance to eliminate the need to measure their influences individually. The investigators can assess the risk of loss in accuracy based on this metric. In the current study, we exclusively utilized US images for steatosis detection. In a real-world federated learning setting, data from each site may exhibit different characteristics. One future research direction involves developing a multimodality model that leverages multiple sources of data to improve the robustness and accuracy of the model. According to the findings of this study, when deploying our framework in a real-world setting, the heterogeneity of data distributions across sites remains a challenge. Each site may possess varying amount of data and exhibit different class distributions. However, the inclusion of participating site from different regions can enhance the performance of the model. In contrast to training a local model, when training a federated learning model in a real-world setting, one must consider both the computation and communication costs. The varying hardware capacities across the sites can greatly affect the training outcome. For example, the straggler effect may occur when a participating site experiences network disconnection or delays. Approaches such as asynchronous federated learning and decentralized networks can be examined to address these challenges.

In conclusion, we have simulated a FL setting for the detection of steatosis using B-mode US images. We have demonstrated the feasibility, performance, and limitations of using FL for steatosis detection. We implemented four simulated FL strategies and evaluated each strategy with four FL algorithms. We also examined the impact of global class imbalance and local heterogeneity on the learning outcome. We found that the quantity skew had a negligible effect on the learning outcome. However, the class distribution skew, particularly when each site possessed samples from only one class, proved to be the most challenging scenario. Furthermore, our findings led to the conclusion that both global class imbalance and local data heterogeneity could have a negative impact on the learning outcome.

### Supplementary Information

Supplementary Information.

## Data Availability

Private dataset (Centre Hospitalier de l’Université de Montréal, Canada): data generated or analyzed during the study are available from the corresponding author by request. Public dataset (Medical University of Warsaw, Poland): data analyzed during the study were provided by a third party. Requests for data should be directed to the provider indicated in the Acknowledgements.
