# Supplementary material for: Simulating federated learning for steatosis detection using ultrasound images
Source: Sci Rep. 2024 Jun 10;14:13253. doi: 10.1038/s41598-024-63969-x (PMC11164945; doi:10.1038/s41598-024-63969-x)
Supplement: Supplementary file 1 — Supplementary Information. [file 41598_2024_63969_MOESM1_ESM.docx]

**Supplementary Information**

**Title:** Simulating Federated Learning for Steatosis Detection Using Ultrasound Images

**Authors:** Yue Qi, Pedro Vianna, Alexandre Cadrin-Chênevert, Katleen Blanchet, Emmanuel Montagnon, Eugene Belilovsky, Guy Wolf, Louis-Antoine Mullie, Guy Cloutier, Michaël Chassé and An Tang

**Table S1.** Diagnostic performance of four federated learning algorithms and four data partition strategies.

| **Data Partition Strategy** | **Metric** | **FedAvg** | **FedAvgM** | **FedYogi** | **FedProx** |
| --- | --- | --- | --- | --- | --- |
| Class distribution skew  (distribution-based) | AUC | 0.87 (0.82, 0.92) | 0.81 (0.79, 0.83) | 0.80 (0.71, 0.89) | 0.87 (0.79, 0.95) |
|  | Sensitivity | 0.81 (0.76, 0.86) | 0.47 (0.04, 0.90) | 0.48 (0.24, 0.72) | 0.84 (0.72, 0.96) |
|  | Specificity | 0.77 (0.70, 0.84) | 0.74 (0.36, 1.00) | 0.85 (0.71, 0.99) | 0.75 (0.67, 0.83) |
|  | Accuracy | 0.79 (0.73, 0.85) | 0.61 (0.59, 0.63) | 0.67 (0.58, 0.76) | 0.79 (0.71, 0.87) |
|  | PPV | 0.78 (0.72, 0.84) | 0.72 (0.57, 0.87) | 0.80 (0.63, 0.97) | 0.77 (0.70, 0.84) |
|  | NPV | 0.80 (0.74, 0.86) | 0.64 (0.48, 0.80) | 0.63 (0.54, 0.72) | 0.83 (0.71, 0.95) |
| Class distribution skew  (quantity-based) | AUC | 0.60 (0.38, 0.82) | 0.50 (0.34, 0.66) | 0.60 (0.50, 0.70) | 0.69 (0.66, 0.72) |
|  | Sensitivity | 1.00 (1.00, 1.00) | 1.00 (1.00, 1.00) | 1.00 (1.00, 1.00) | 0.97 (0.90, 1.00) |
|  | Specificity | 0.00 (0.00, 0.01) | 0.00 (0.00, 0.00) | 0.00 (0.00, 0.00) | 0.11 (0.00, 0.34) |
|  | Accuracy | 0.50 (0.50, 0.50) | 0.50 (0.50, 0.50) | 0.50 (0.50, 0.50) | 0.54 (0.46, 0.62) |
|  | PPV | 0.50 (0.50, 0.50) | 0.50 (0.50, 0.50) | 0.50 (0.50, 0.50) | 0.52 (0.47, 0.57) |
|  | NPV | 0.33 (0.00, 0.99) | 0.00 (0.00, 0.00) | 0.00 (0.00, 0.00) | 0.26 (0.00, 0.77) |
| Quantity skew | AUC | 0.92 (0.91, 0.93) | 0.82 (0.79, 0.85) | 0.78 (0.72, 0.84) | 0.92 (0.90, 0.94) |
|  | Sensitivity | 0.88 (0.71, 1.00) | 0.85 (0.78, 0.92) | 0.60 (0.17, 1.00) | 0.89 (0.79, 0.99) |
|  | Specificity | 0.77 (0.62, 0.92) | 0.63 (0.44, 0.82) | 0.73 (0.47, 0.99) | 0.81 (0.76, 0.86) |
|  | Accuracy | 0.82 (0.79, 0.85) | 0.74 (0.67, 0.81) | 0.66 (0.52, 0.80) | 0.85 (0.82, 0.88) |
|  | PPV | 0.80 (0.72, 0.88) | 0.70 (0.61, 0.79) | 0.68 (0.56, 0.80) | 0.83 (0.80, 0.86) |
|  | NPV | 0.89 (0.77, 1.00) | 0.81 (0.79, 0.83) | 0.70 (0.51, 0.89) | 0.89 (0.80, 0.98) |
| Source-based partition | AUC | 0.93 (0.92, 0.94) | 0.81 (0.73, 0.89) | 0.80 (0.74, 0.86) | 0.88 (0.81, 0.95) |
|  | Sensitivity | 0.88 (0.76, 1.00) | 0.55 (0.11, 0.99) | 0.78 (0.64, 0.92) | 0.88 (0.82, 0.94) |
|  | Specificity | 0.75 (0.65, 0.85) | 0.80 (0.60, 1.00) | 0.78 (0.63, 0.93) | 0.72 (0.69, 0.75) |
|  | Accuracy | 0.82 (0.80, 0.84) | 0.68 (0.56, 0.80) | 0.78 (0.75, 0.81) | 0.80 (0.79, 0.81) |
|  | PPV | 0.79 (0.74, 0.84) | 0.78 (0.69, 0.87) | 0.79 (0.70, 0.88) | 0.76 (0.75, 0.77) |
|  | NPV | 0.88 (0.77, 0.99) | 0.69 (0.51, 0.87) | 0.79 (0.72, 0.86) | 0.86 (0.80, 0.92) |

Note.— The models were trained for 1 epoch per round. *AUC* = area under the receiver operating characteristic curve, *NPV* = negative predictive value, and *PPV* = positive predictive value. Data in parentheses are 95% CIs.


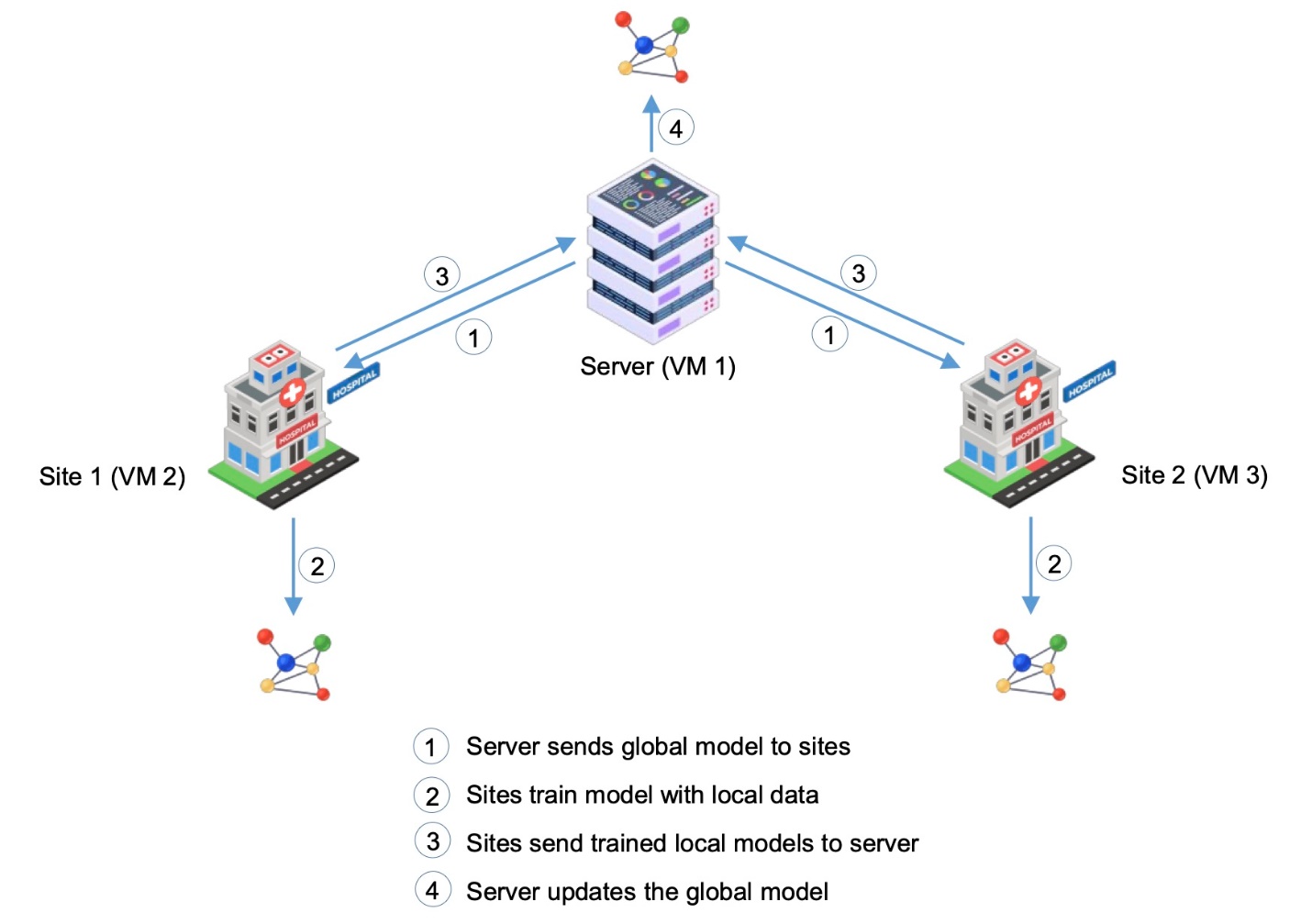


**Figure S1.** Design of the federated learning simulation environment. Typically, a four-step process is followed for each training round. Initially, the server transmits the most up-to-date global model to the sites. Subsequently, the sites train the received model using their local data. Afterwards, the sites send the trained local model back to the server. Finally, the server updates the global model based on the received contributions. *VM =* virtual machine. Credit: server, site and model icons, Flaticon.com.


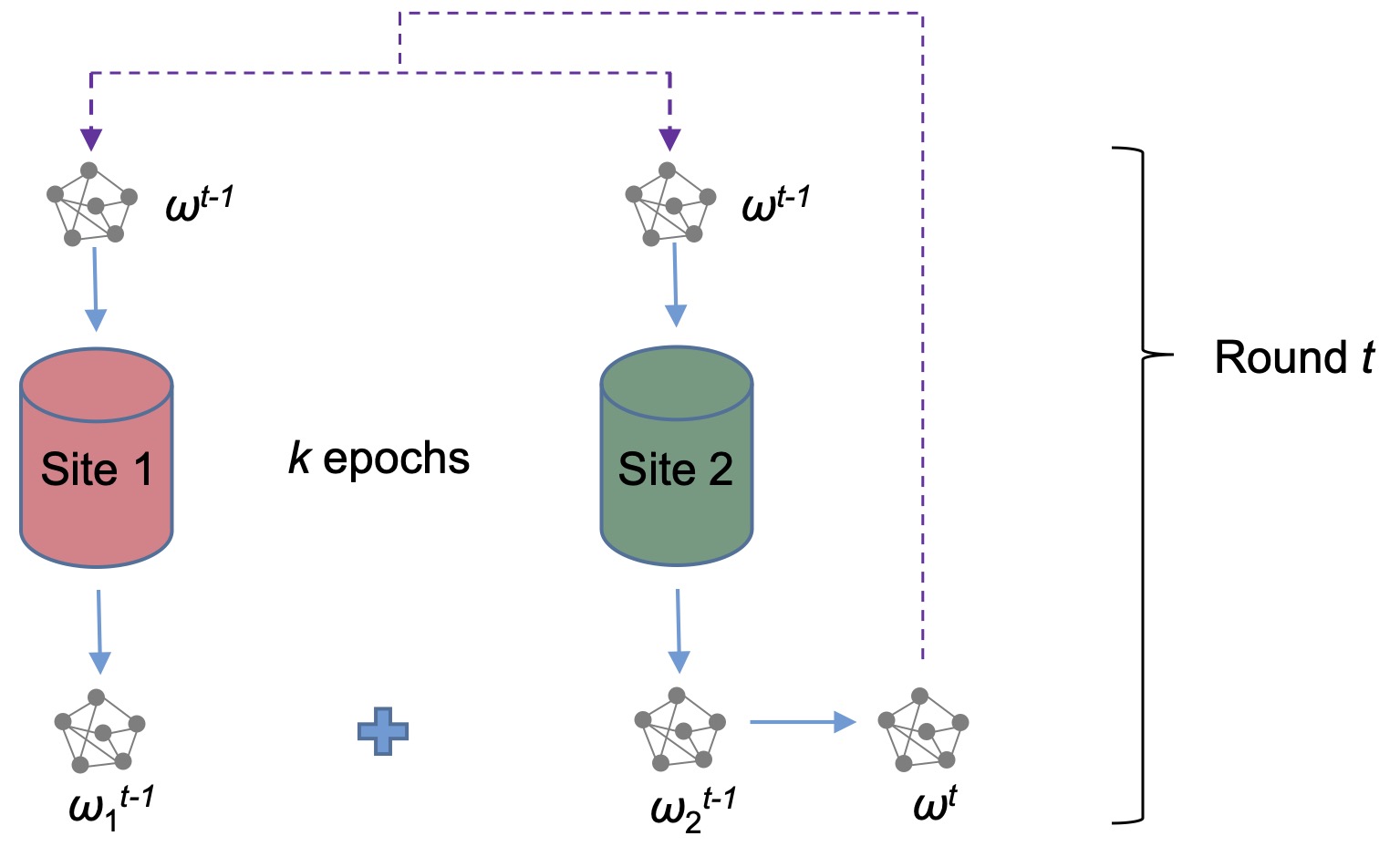


**Figure S2.** FedAvg training round: (1) server distributes the weights of the global model to each site, (2) sites train the model with local data, (3) sites transmit the weights of trained model to the server and (4) server aggregates the received weights.
